# Fibroid Degeneration of the Vermiform Appendix

**Published:** 1910-03-19

**Authors:** 


### SPECIAL ARTICLES.

### FIBROID DEGENERATION OF THE VERMIFORM APPENDIX.

There is a variety of appendicular lesion which has hitherto received little attention; this is obliterative fibrosis, which may convert the organ into a solid cord, and thus bring about spontaneously protection against microbial infection. It is a subject upon which Mr. Robert T. Morris, Professor of Surgery at the New York Post-graduate Medical School and Hospital, expresses views that are worthy of consideration. He says that perhaps the time has come to classify four separate and distinct kinds of appendicitis, namely: (1) Cases of fibroid degeneration of the appendix; (2) appendicitis with intrinsic infection; (3) congestive appendicitis; (4) appendicitis with extrinsic infection.

For practical use he holds that this classification is convenient, even though it may not be scientifically accurate. It may be urged directly that the four kinds all merge into one another. Professor Morris himself formerly thought so, and that cases 1, 3, and 4 commonly merge into cases 2. Observations directed towards this particular point during fifteen years, however, have tended to disprove that idea, and to separate the different kinds of appendicitis from each other more and more.

Into Class 1 he puts cases with fibroid degeneration of the appendix, which he thinks are the most common and yet the most frequently overlooked. Fibroid degeneration of the appendix, he says, probably sends a much larger number of patients to the doctor than does any other type of appendix trouble. It is relatively mild in its effects, so that it is clearly more commonly seen by general practitioners than by specialists or consultants. Fibroid degeneration of the appendix may begin in childhood, but it is seldom productive of symptoms before adult life has been reached, and it is apparently most troublesome in individuals between the ages of 30 and 50. Senn first called attention to the subject in an article published under the title of " Appendicitis Obliterans " in 1894. In 1902 Ribbert, in his " Lehrbuch der speciellen Pathologie," describes the appendix as an organ which, by reason of its uselessness, begins to undergo involution at an early period. With a lumen completely pervious in youth he regards it as gradually becoming obliterated, so that after sixty years of age over 50 per cent. of cases show total or partial obliteration. Ribbert believes that it is the gradual effacement of the lumen which is largely responsible for the various forms of inflammation of the organ. Morris's observations, on the other hand, lead to quite the contrary conclusion; it is his belief that with gradual disappearance of the structures which are involved in acute infective processes the tendency to such processes disappears. Further, that an appendix which is undergoing fibroid degeneration seems to remain in a condition of irritation which calls out constant protection against infection, and that the patients who suffer most often from the symptoms attending fibroid degeneration of the appendix are really those who are best protected against infective invasions of the organ. His conclusion has been disputed by surgeons who have called attention to the fact that fibrous tissue is found in appendices which they have removed, and which have been at the time the seat of acute intrinsic infection. His answer is that in this particular class of cases the fibrous tissue appears to represent scar tissue resulting from the distinctive inflammation of previous acute attacks of infection. Such, at least, has been the history in his cases frequently enough to allow him still to keep the scarred appendices out of the class of fibroid degeneration of the appendix.

The reason for the belief that the two kinds of appendices can be kept apart is this. With fibroid degeneration there is gradual replacement of all its structures; but nerve filaments persist longest of all, and these irritated by the contraction of the hyperplastic connective tissue cause persistent local and reflex disturbance. The scarred appendices, on the other hand, lose nerve filaments along with other structures at the time of the acute infection and its general toxic destruction of cells. Consequently in the intervals between attacks the scarred appendices do not cause the set of symptoms that are so persistent and so characteristic in cases of fibroid degeneration of the appendix. Mr. Morris says that he would prefer to believe, with Ribbert, that fibroid degeneration of the appendix is a menace, and provocative of acute infective attacks, because it would then allow him to advise operation in practically all of these cases; but from the point of view he holds he regards it as best to advise against operation in most of the fibroid cases, when there is no disturbance beyond some sense of discomfort in the appendicular region.

Many people with fibroid appendices seem not to suffer in any way. A certain number experience merely a sense of discomfort in the right iliac fossa. They are inclined to press upon that region with the hand or to lean against the edge of a table or chair, and they go to a medical man to ask if they have appendicitis. A very large class includes those patients who, in addition to a sense of discomfort have occasional attacks of sharp pain of varying duration in the right iliac fossa, and they often suffer one or other of the various disturbances of function of the bowel. Fibroid degeneration of the appendix is perhaps the most common single cause for so-called intestinal dyspepsia; at least, it seems to be the most common overlooked cause. Fibroid degeneration of the appendix may be a potent factor in certain cases of neurasthenia. In making this statement Mr. Morris wishes it to be clearly understood that he is quite familiar with the complex nature of neurasthenia, and knows that it is not often that any one disease or defect stands alone as its causative factor. A single disease or defect commonly enough stands alone as a precipitating factor, however, and it is in this respect that the appendix undergoing fibroid degeneration may play an important part. One is familiar with the patient who goes about from doctor to doctor trying to obtain a definite diagnosis referable to the appendix, and the different opinions that he obtains are apt to leave him much confused. The most common diagnosis he obtains is "chronic inflammation of the appendix" or "chronic appendicitis"; and the most common advice is perhaps that he ought to have the appendix removed, on the ground that acute appendicitis is liable to supervene at any time. Such a diagnosis does not indicate the real cause for the chronic irritation, and the advice is unwarranted, according to Mr. Morris's views upon the pathology of the condition.

Examination of appendices that are undergoing fibroid degeneration shows various degrees of replacement of the normal structures of the appendix by fibrous tissue. There is apt to be a rather even replacement, involving lymphoid and submucous tissues primarily and beginning at the distal end. The mucous coat disappears next, then the muscular coat, and in some cases the peritoneal coat in part or as a whole. The larger blood-vessels are compressed and obliterated at an early date, but the nerve filaments seem to resist pressure atrophy for a long time. Many years are required for completion of the changes; some patients suffer enough to make it worth while to have the appendix removed because of the influence of the changes in it on the general health rather than because of danger from infection of the appendix leading to acute appendicitis.

The diagnosis of fibroid degeneration of the appendix can usually be made without much difficulty. These fibroid appendices feel distinctly harder than normal as they slip from under the palpating fingers. Tenderness of the appendix may not be a very marked feature, and it is rather less in degree than one expects to find in connection with the history of discomfort, pain, or reflex disturbances. The chief diagnostic point consists in finding a point of tenderness at the site of the right lumbar ganglia, to be found by pressing upon the abdominal wall at a point about an inch and a half to the right of the umbilicus, carrying the fingers well down toward the right side of the spinal column. If one finds hyperæsthesia at the site of the right lumbar ganglia alone, it is very probable that the appendix is the source of irritation. If, however, the left lumbar ganglia are equally sensitive, one should look to the pelvis for the seat of mischief; whilst, if neither right nor left lumbar ganglia are hypersensitive, appendicular irritation is unlikely.

There is little need to say more than a word or two about each of the other three classes of appendicular trouble distinguished by Professor Morris.

Class 2, intrinsic infective appendicitis, he regards as due to the invasion of anæmic areas in the appendix by intestinal bacteria, the anæmia being usually a compression anæmia caused by swelling of the inner structures of the organ within a tight outer sheath. The cause for the swelling is of less consequence in itself than it is as a producer of local anæmia by compression.

Class 3, congestive appendicitis, seems to be a benign condition occurring in association with congestion of other abdominal organs; and even though the appendix is sometimes distended with serous exudate to the point of becoming anæmic, the congestive process takes place so slowly that protective factors seem to be called out, and there is little danger of these cases merging into Class 2.

Class 4, appendicitis due to extrinsic infection, is found in cases where such diseases as tuberculosis or cancer involve the appendix. In these cases also one seems to have some protective mechanism set to work during the slow advance of the disease so as to guard against acute attacks.

Whether we agree with Professor Morris in his views upon fibroid degeneration of the appendix or not—particularly in respect of his contention that, far from rendering the patients liable to acute exacerbations, it protects them against acute infective appendicitis—we cannot but reflect upon what he says. The consulting surgeon is very apt to see so many cases requiring appendicectomy, that he begins to teach that every patient who has any appendicular symptoms ought to have his appendix out. The general practitioner knows that any number of appendicular cases do perfectly well without any operation at all. It is a good thing to think deliberately upon the subject, and it is also good to hear a professor of surgery not only introducing a term such as "fibroid degeneration of the appendix," which conveys so very different a meaning to "chronic appendicitis," but also insisting that these cases, which are really numerous, are not in danger and do not require appendicectomy.

